# Greek College Students and Psychopathology: New Insights

**DOI:** 10.3390/ijerph120504709

**Published:** 2015-04-29

**Authors:** Konstantinos Kontoangelos, Sofia Tsiori, Kalliopi Koundi, Xenia Pappa, Pavlos Sakkas, Charalambos C. Papageorgiou

**Affiliations:** 1 1st Department of Psychiatry, Athens University Medical School, Eginition Hospital, 74 Vas. Sofias Ave., Athens 11528, Greece; E-Mails: sofiatsiori@gmail.com (S.T.); kkoundi@med.uoa.gr (K.K.); xpappa@med.uoa.gr (X.P.); psakkas@med.uoa.gr (P.S.); cpapage@eginitio.gr (P.C.C.); 2 University Mental Health Research Institute, 27 Soranou tou Efesiou str., Athens 11527, Greece

**Keywords:** college students, psychopathology, depression, personality, disorder, somatization

## Abstract

*Background*: College students’ mental health problems include depression, anxiety, panic disorders, phobias and obsessive compulsive thoughts. *Aims*: To investigate Greek college students’ psychopathology. *Methods*: During the initial evaluation, 638 college students were assessed through the following psychometric questionnaires: (a) Eysenck Personality Questionnaire (EPQ); (b) The Symptom Checklist-90 (SCL-90); (c) The Beck Depression Inventory (BDI); (d) State-Trait Anxiety Inventory (STAI). *Results*: State anxiety and trait anxiety were correlated, to a statistically significant degree, with the family status of the students (*p* = 0.024) and the past visits to the psychiatrist (*p* = 0.039) respectively. The subscale of psychoticism is significantly related with the students’ origin, school, family status and semester. The subscale of neuroticism is significantly related with the students’ school. The subscale of extraversion is significantly related with the students’ family psychiatric history. Students, whose place of origin is Attica, have on average higher scores in somatization, phobic anxiety and paranoid ideation than the other students. Students from abroad have, on average, higher scores in interpersonal sensitivity and psychoticism than students who hail from other parts of Greece. The majority of the students (79.7%) do not suffer from depression, according to the Beck’s depression inventory scale. *Conclusions*: Anxiety, somatization, personality traits and depression are related with the students’ college life.

## 1. Introduction

Students’ mental health problems include depression, anxiety, panic disorders, phobias and obsessive compulsive thoughts [1,2]. During college years, students are confronted with academic, social and personal needs. These needs may shift along with changes in age, experience, socioeconomic status, gender, race or ethnicity and social trends. Researchers consent on the need for an accurate, regular assessment of college student needs [3].

Mental disorders are as prevalent among college students as same-aged nonstudents [4] and these disorders appear to be increasing in number and severity [5,6]. Mental health among college students presents not only a growing concern but also an opportunity, because of the large number of people who could be reached during an important period of life. More than 65% of American high school graduates continue with higher education [7]. Mental disorders account for nearly one-half of the disease burden for young adults in the United States [8] and most lifetime mental disorders have first onset by the age of 24 years [9]. The college years represent a developmentally challenging transition to adulthood, and untreated mental illness may have significant implications on academic success [10], productivity [11], substance use [12,13] and social relationships [14].

Within the college population, certain subgroups have a significantly larger prevalence of mental health problems, which is consistent with studies of the general population. Male undergraduates are at a higher risk for suicide, but female students are more likely to screen positive for major depression and anxiety disorders [15]. Students from lower socioeconomic backgrounds are at a higher risk for depressive and anxiety symptoms [16]. Poor mental health is also more common among students with relationship stressors [17], low social support [18], or victimization by sexual violence [19]. Considering the above, the aims of the present study were to examine the relationship among depression, anxiety, personality and psychosomatic symptoms.

## 2. Methods

The sample was selected randomly at the university campus. During the initial evaluation, 638 college students were assessed through the following psychometric questionnaires.

### 2.1. Psychometric Questionnaires

(i). Psychometric Personality scale of extraversion, neuroticism, psychotism (Eysenck Personality Questionnaire, EPQ) (Eysenck 1975) [20]. The Eysenck personality questionnaire consists of 84 entries evaluated by the patient with a yes or no answer. The purpose of this questionnaire is to explore four dimensions of personality: psychotism (P), neuroticism (N) extraversion (E) and lying (L). The scales N and L are of particular clinical interest. The N scale is the best studied and is associated with a clinical diagnosis of neurosis or oral personalities according to psychoanalytic terminology. The E scale corresponds roughly to histrionic personalities. The P scale corresponds to obsessive-compulsive personalities and is unrelated to psychosis. The L scale was introduced later in an attempt to measure the extent which subjects were deliberately attempting to control their scores (Dimitriou 1986) [21].

(ii). The Symptom Checklist-90 (SCL-90) [22]. The questionnaire is self-completed and measures 9 psychopathology parameters (as many as its subscales), which are: (1) somatization; (2) depression; (3) anxiety; (4) phobic anxiety; (5) obsessive compulsive; (6) paranoid ideation; (7) psychoticism; (8) hostility; (9) interpersonal sensitivity. The questionnaire includes 90 questions in total. All entries are rated from 0 to 4, giving a total score of 360. The scale is used to extrapolate 3 aggregate indexes: (a) the general severity index; (b) the positive symptoms distress index; (c) the positive symptoms total. A weighted Greek version is available. The general symptomatic index can be computed from the SCL-90 by simple arithmetic by adding all the raw factor scores and divide by 90. The positive Symptom Distress Level is the average level of distress of those symptoms out of 90, to which the patient indicates any degree of distress. The positive symptom total is a number of symptoms out of 90, to which the patient indicates any degree of distress [23].

(iii) The Beck Depression Inventory (BDI) is a 21-question multiple-choiceself-assessment report inventory, one of the most widely used instruments for measuring the severity of depression. Its development marked a shift among health care professionals, who had until then viewed depression from a psychodynamic perspective, instead of it being rooted in the patient's own thoughts. In its current version, the questionnaire is designed for individuals aged 13 and over, and is composed of items related to symptoms of depression such as hopelessness and irritability, cognitions such as guilt or feelings of being punished, as well as physical symptoms such as fatigue, weight loss and lack of interest in sex. Abbreviation has been stated, when the test is scored, a value of 0 to 3 is assigned for each answer and then the total score is compared to a key to determine the depression severity. The standard cut-offs are as follows: 0–9: indicates minimal depression, 10–18: indicates mild depression, 19–29: indicates moderate depression, 30–63: indicates severe depression [24].

(iv). The State-Trait Anxiety Inventory (STAI) is a psychological inventory based on a 4-point Likert scale and consists of 40 questions on a self-report basis. The STAI measures two types of anxiety: state anxiety or anxiety about an event and trait anxiety, or anxiety level as a personal characteristic. Higher scores are positively correlated with higher levels of anxiety. Its most current revision is Form Y which is offered in 12 languages. Their goal in creating the inventory was to create a set of questions that could be applied to assessing different types of anxiety. This was a new development because all other questionnaires focused on one type of anxiety at a time. Spielberger also created other questionnaires, like the STAI, that assessed other emotions. These are the State-Trait Anger Scale (STAS), State-Trait Anger Expression Inventory (STAXI), and the State-Trait Anxiety Inventory for Children (STAIC). The following cut-off scores have been proposed: 20–39 low stress, 40–59 moderate stress, 60–80 high stress [25].

### 2.2. Statistical Analysis

Descriptive statistics include means and standard deviations for normally distributed continuous variables and quartiles for non-normally distributed variables. Percentages are given for categorical variables. Associations among groups of students and the scales of STAI are explored through the Student’s *t*-test and linear regression analysis. Scales based on the sum of binary questions (such as the scales of EPQ) are assumed to follow the binomial distribution and thus binomial generalized linear models were used to explore the corresponding relations. Over dispersion problems are handled by using the quasi binomial distribution family. Robust linear regression analysis was used to examine the relations among the subscales of SCL-90 and the students’ characteristics. Multiple logistic regression analysis is performed to examine the association between the students’ background and the Beck’s scale. The statistical tests presented here are two-tailed and compared with the statistical level of 5%.

The statistical package SPSS 21 was used to perform descriptive statistics, *t*-test, linear and logistic regression analysis. Binomial generalized linear models were performed on SPSS, however, the GLM package in R 2.15.1 was also used to handle over-dispersion problems. Robust linear regression analysis was implemented in STATA.

## 3. Results

### 3.1. Demographic Characteristics

Demographic characteristics for the students who participated in this study are summarized in Table 1. The majority of the students are females (63.38%) and their ages vary from 18 to 37 years old. Almost half of the students are studying at the School of Science (48.98%), and 15.34% are studying at the School of Health Science, 14.40% at the School of Philosophy and 13.46% at the School of Law and Economics and Political Science. In the analysis that follows, the School of Theology is merged with the other departments, since the corresponding percentage is low.

**Table 1:** Demographic characteristics of the sample.

Category		Frequency	Percent	Job	Frequency	Percent
Female		405	63.38	No	529	82.79
Male		234	36.62	Yes	110	17.21
Total		639		Total	639	
School				Residence		
Theology		7	1.10	Family	277	43.42
Law, Economics and Political Science		86	13.46	Brothers/Sisters	72	11.29
Health		98	15.34	Flatmates	27	4.23
Philosophy		92	14.40	Students’ residence	157	24.61
Science		313	48.98	Alone	95	14.89
Other departments		43	6.73	Other	10	1.57
Total		639		Total	638	
Income				Origin		
No		321	50.39	Attica	308	48.28
0–3000		126	19.78	Urban areas	205	32.13
3001–8200		96	15.07	Hail from other parts of Greece	70	10.97
8201–12,600		42	6.59	Rural areas	26	4.08
>12,601		52	8.16	Abroad	29	4.55
Total		637		Total	638	
Semester				BMI		
Undergraduate		460	72.33	Underweight	47	7.83
About to graduate		164	25.79	Normal	456	76.00
Postgraduate		12	1.89	Overweight	86	14.33
Total		636	Obese	11	1.83	
Family status				Total	600	
Single		594	93.10	Traumatic death		
Married		39	6.11	Nothing	357	84.20
Cohabitation		2	0.31	Committed suicide	13	3.07
Separated		2	0.31	Attempted suicide	22	5.19
Divorced		1	0.16	Violent dealth	32	7.55
Total		638		Total	424	
Health problem				Family history		
No		578	90.45	No	553	86.68
Yes		61	9.55	Yes	85	13.32
Total		639	Total	638		
Past visits				Drugs		
No		351	82.78	No	403	95.05
Yes		73	17.22	Yes	21	4.95
Total		424		Total	424	
**Age**	***n***	**Minimum**	**Maximum**	**Mean**	**Median**	**Std. Variation**
	638	18.00	37.00	21.72	21.0	2.79

Most of the students are still staying with their families during their studies (43.42%), whereas, the percentage of students who are staying in students’ dormitory is 24.61%. In the forthcoming analysis, this variable is categorized into 3 groups, indicating whether students are staying with their families, in students’ dormitory or somewhere else.

Almost half of the students originally come from Attica (48.28%), 47.18% who hail from the other parts of Greece and 4.55% from abroad. The majority of the students who visited the unit are undergraduate students who have not completed the fourth year of their studies (72.33%), whereas, 25.79% of the students are undergraduates who have not completed their studies after the fourth year. There is also a small percentage of postgraduate students (1.89%).

Almost half of the students declared that they do not have their own income, and 82.79% of the students are not working during their studies. The majority of the students in our sample are single (93.10%). The variables of income and family status are treated as binary (yes/no and married/not married) in the following analysis.

The Body Mass Index (BMI) is also calculated for the students, where it turns out that the majority of the students (76%) have normal weight, 16.16% are overweight or obese and 7.83% are underweight. In terms of their medical history, the majority of the students do not have a health problem, 17.22% of them have visited the unit in the past and 13.32% of the students have a psychiatric family history. There is a percentage of students (15.8%) who declared that they have experienced a traumatic death in their family and almost 5% of the students who visited the unit are addicted to drugs.

### 3.2. State-Trait Anxiety Inventory (STAI)

Descriptive statistics for the scales of State-Trait anxiety inventory (STAI) are displayed in Table 2. We examined the relation of the STAI scales with each of the students’ characteristics and found out that the state anxiety is significantly related with the family status of the students and the trait anxiety is significantly related with the past visits to the psychiatrist (Table 3). Students who are married have on average higher scores on state anxiety scale than students who are not married (including single, divorced, separated or in cohabitation). Students who have visited the University psychiatric unit in the past have on average higher scores on trait anxiety scale than those who do not.

**Table 2:** Descriptive statistics for State-Trait anxiety inventory (STAI) scale.

STAI	*n*	Minimum	Maximum	Mean	Std. Deviation
Trait Anxiety	639	20.00	73.00	44.28	10.11
State Anxiety	639	20.00	79.00	44.15	11.59

**Table 3:** *T*-test for State-Trait anxiety inventory (STAI) scales with family status and past visits.

STAI		*n*	Mean	Std. Deviation	*p*-Value
State Anxiety	**Family Status**				0.024
	Not married	599	43.88	11.64	
	Married	39	48.21	10.29	
Trait Anxiety	**Past Visit**				0.039
	No	351	43.52	9.82	
	Yes	73	46.15	10.28	

The relations between the STAI scale and the students’ characteristics were further examined through linear regression analysis. The final model was selected through forward stepwise procedure, whereas, no significant interactions were found among the variables. It turns out that both state and trait anxiety scales vary among the schools that students are studying at, the students’ origin and their health status (Table 4).

Students who hail from other parts of Greece are expected to score on average lower on STAI scale than the students from Attica while the other variables in the model are held constant. On the other hand, students from abroad are expected to score higher than students from Attica. Both state and trait scales differ significantly between the students who hail from other parts of Greece and Attica, whereas, only the state scale differs significantly between the students from abroad and Attica.

Students from the school of health science are expected on average to have lower scores on STAI scales than students from all other departments (including law, philosophy and science) given that the remaining variables do not change. The differences are significant for both scales. Students who reported that they have health problems are expected to have a higher score on STAI scales. Besides that, males are expected to have lower scores than females.

**Table 4:** Linear regression coefficients for State and Trait anxiety scales.

Demographic	State Anxiety		Trait Anxiety	
	B	*p*-Value	B	*p*-Value
**Origin**				
Hail from other parts of Greece	−2.95	0.002	−2.58	0.002
Abroad	4.71	0.033	2.37	0.218
**School**				
Law, Economics and Political Science	4.36	0.010	2.41	0.100
Philosophy	3.38	0.041	5.12	<0.001
Science	2.94	0.025	3.08	0.007
Other	5.02	0.011	4.16	0.015
**Health problem**				
Yes	3.15	0.040	3.04	0.023
**Gender**				
Male	−3.32	<0.001	−3.18	<0.001
**Age**	0.32	0.053	0.23	0.108

### 3.3. Results from Eysenck Personality Questionnaire (EPQ)

Descriptive statistics for EPQ scales are summarized in Table 5. The EPQ questionnaire consists of polar questions (yes/no) and from now and on we define as “*positive answer*” the answer “*yes*” in the questions with positive aspect and the answer “*no*” in those with negative connotation. Therefore, each subscale is assumed to follow the binomial distribution with number of trials equal to the number of questions (24 for psychoticism, 22 for neuroticism and 19 for extraversion and lie) and the probability of success corresponds to the probability of positive answer. We therefore use binomial generalized linear models to examine the relations between the EPQ subscales and the students’ characteristics. The final models for each subscale are summarized in Table 6. Students’ age and gender are included in the models, whereas, no interaction terms were found to be significant. The interpretation of the coefficients that follows is made under the assumption that all the other variables remain constant.

**Table 5:** Descriptive statistics for Eysenck Personality Questionnaire (EPQ) scales.

EPQ	*n*	Minimum	Maximum	Mean	Std. Deviation	Percentiles		
						25%	Median	75%
Psychotism	639	0	16.00	2.06	2.20	1.00	1.00	2.00
Neuroticism	639	0	22.00	11.83	4.84	8.00	12.00	15.00
Extraversion	639	0	19.00	13.38	4.27	11.00	14.00	17.00
Lie	639	0	18.00	8.68	3.45	6.00	9.00	11.00

The subscale of psychoticism is significantly related to the students’ origin, school, family status, semester and the variable indicating if they are working or not. In particular, students who hail from other parts of Greece have lower odds of giving positive answers to the questions related with the psychoticism than students who come from Attica. On the other hand, students from abroad have higher odds than students from Attica. Students who are studying in the school of Philosophy have lower odds of giving positive answers than students in all the other departments. Particularly, students in the schools of Law, Economics and Political Science and in Science have significantly higher odds than the students of Philosophy. Students who are married have 50% higher probability of giving positive answers than the students who are not married. Students who are working during their studies have higher probability of scoring higher in psychoticism than students who are not working. Finally, students who are studying for more than four years have higher odds than those who have not completed the 4th year of their studies. On the other hand, postgraduate students have lower odds of giving positive answers than undergraduate students.

**Table 6:** Binomial generalized lineal models for each subscale of Eysenck Personality Questionnaire (EPQ).

Demographic		Psychoticism	Neuroticism	Extraversion	Lie
		OR	OR	OR	OR
**Origin** (Ref. level: Attica)	Hail from other parts of Greece	0.69 ^***^			
	Abroad	1.77 ^***^			
**School** (Ref. level: Philosophy)	Law, Economics and Political Science	1.60 ^***^	1.07		
	Health	1.23	0.85		
	Science	1.34 ^**^	0.75 ^**^		
	Other	1.20	0.78		
**Family status** (Ref. level: Not married)	Married	1.51 ^**^			
**Job** (Ref. level: No)	Yes	1.30 ^**^		1.38 ^***^	
**Semester** (Ref. level: Undergraduate)	>4th year	1.32 ^**^			
	Postgraduate	0.46 ^*^			
**Residence** (Ref. level: Family)	Students’ residence				1.48 ^***^
	Other				1.24 ^**^
**Income** (Ref. level: No)	Yes		1.24 ^**^		
**Past visits** (Ref. level: No)	Yes		1.31 ^**^		
**Family history** (Ref. level: No)	Yes			0.77 ^**^	
**Drugs** (Ref. level: No)	Yes				0.68 ^**^
**Traumatic Death** (Ref. level: No)	Yes				0.82 ^**^
**Gender** (Ref. level: Female)	Male		0.77^**^		
**Age**				0.96 ^**^	
Pearson Chi-Square/df		2.19 ^***^	3.89 ^**^	4.53 ^***^	2.39 ^***^

^***^ *p*-value < 0.001; ^**^ *p*-value < 0.05; ^*^ *p*-value < 0.10

The subscale of neuroticism is significantly related with the students’ school, and the variables indicating whether they have their own income and visited the psychiatric unit in the past. Students who are studying science have lower probability of scoring high in neuroticism than students in philosophy. Students who have their own income have higher odds to give positive answers than students without any income. Besides that, students who have visited the psychiatric unit in the past have 31% higher odds of giving positive answers than the students who have not visited the unit in the past. It turns out that males have significantly lower odds than females.

The subscale of extraversion is significantly related with the students’ family psychiatric history and whether they are working or not. Students who are working have higher odds of scoring higher in this scale than students who are not working. Students with a psychiatric family history have lower odds in giving positive answers to the questions concerning the extraversion than the students without a family history. Finally, older students tend to have lower odds than younger students.

The subscale of lie is significantly related to the students’ residence, their addiction to drugs and the occurrence of a traumatic death in their family. Students who are staying in students’ dormitory have almost 50% higher odds to give positive answers to the questions related with the lie scale than students who are staying with their family. Students who have other residence (including staying with brother/sisters, flat mates or alone) have 24% higher odds to give positive answers than the students who are staying with their family. Students who are addicted to drugs have lower odds than students who are not drug addicts. Finally, students who have experienced a traumatic death in their family have lower odds of scoring high in lie scale than students who do not have such experience.

### 3.4. Results from SCL-90

Descriptive statistics for the scales of SCL-90 are given in Table 7. Robust linear regression models for each SCL-90 subscale were selected through the backward procedure and are displayed in Table 8. Students who hail from other parts of Greece are expected to have higher scores on somatization and interpersonal sensitivity and lower scores on depression scale than students who are coming from Attica. Students from abroad are expected to have higher scores on obsessive compulsive, phobic anxiety, paranoid ideation, psychoticism and lower scores on somatization and interpersonal sensitivity than students from Attica.

**Table 7:** Descriptive statistics for SCL-90 scales.

SCL-90	*n*	Mean	Std. Deviation	Percentiles		
				25%	Median	75%
Somatization	639	6.69	7.93	1.00	3.00	10.00
Obsessive-Compulsive	639	14.15	7.65	8.00	14.00	20.00
Interpersonal Sensitivity	639	7.41	7.59	0.00	6.00	13.00
Depression	639	15.70	10.01	8.00	14.00	22.00
Anxiety	639	9.66	7.58	4.00	8.00	14.00
Hostility	639	6.21	5.07	2.00	5.00	9.00
Phobic anxiety	639	4.11	4.49	0.00	3.00	6.00
Paranoid Ideation	639	7.09	5.01	3.00	6.00	10.00
Phychoticism	639	8.11	7.10	3.00	6.00	12.00

**Table 8:** Robust linear regression model for each scale of SCL-90.

Demographic		Somatization	Obsessive-Compulsive	Interpersonal Sensitivity	Depression	Anxiety	Hostility	Phobic Anxiety	Paranoid Ideation	Psychoticism
**Origin** (Ref. level: Attica)	Hail from other parts of Greece	1.07 ^**^	−0.81	2.06 ^**^	−2.03 ^**^			−0.10	−0.49	−0.63
	Abroad	−1.95 ^**^	3.23 ^**^	−3.09 ^**^	1.82			2.45 ^***^	2.70 ^**^	3.27 ^**^
**School** (Ref. level: Philosophy)	Law, Economics and Political Science	−1.59 ^**^	−1.39	−2.46 ^**^	−2.37			−0.14	−1.19	−1.26
	Health	−0.60	−3.52 ^**^	−1.71	−5.49 ^***^			−1.19 ^**^	−2.81 ^**^	−3.26 ^**^
	Science	−1.03	−0.37	−2.36 ^**^	−1.73			−0.33	−0.98	−1.41 ^*^
	Other	−2.19	−0.55	−3.00 ^**^	−2.46			−0.08	−0.72	−1.44
**Family status** (Ref. level: Not married)	Married			−3.00 ^**^				1.42 ^**^		2.40 ^**^
**Semester** (Ref. level: Undergraduate)	>4th year	−1.72 ^**^								
	Postgraduate	1.30								
**Residence** (Ref. level: Family)	Students’ residence			1.17		−1.64 ^**^	−1.01 ^**^			
	Other			−2.61 ^**^		−0.72	−0.89 ^*^			
**BMI** (Ref. level: Normal)	Underweight	1.57 ^**^				3.30 ^**^		1.20 ^**^		2.17 ^**^
	Overweight/obese	0.55				0.98		0.17		0.90
**Health Problem** (Ref. level: No)	Yes									
					4.01 ^**^	2.77 ^**^	1.64 ^**^		1.38 ^*^	3.01 ^**^
**Gender** (Ref. level: Female)	Male	−1.35 ^**^	−1.71 ^**^	−0.87	−3.23 ^***^	−1.76 ^**^	−0.16	−0.86 ^**^	−0.05	−0.29
**Age**		−0.19 ^**^	0.09	−0.26 ^**^	0.05	−0.03	−0.09	−0.16 ^**^	−0.09	−0.20 ^*^

^***^ *p*-Value < 0.001; ^**^ *p*-value < 0.05; ^*^ *p*-value < 0.10.

Students who are studying Philosophy are expected to have higher scores on all the scales as shown in Table 8. In particular, the expected scores of somatization and interpersonal sensitivity of the students from the school of philosophy are significantly greater than the students who are studying low, economics and political science. The students from health school are expected to score significantly lower in obsessive compulsive, depression, phobic anxiety, paranoid ideation and psychoticism scales than the students in Philosophy.

Students who are married are expected to score significantly lower on the scale of interpersonal sensitivity and higher on phobic anxiety and psychoticism than students who are not married. Undergraduate students are expected to score significantly higher on the scale of somatization than students who are still studying after the fourth year. Underweight students are expected to score higher on the scales of somatization, anxiety, phobic anxiety and psychoticism than students with normal weight. Students who have been diagnosed with a health problem are expected to score significantly higher on the scales of depression, anxiety, hostility and psychoticism than healthy students.

Females are expected to score higher on somatization, obsessive compulsive, depression, anxiety and phobic anxiety than males. Finally, students’ age is significantly correlated with the scales of somatization, interpersonal sensitivity, phobic anxiety and psychoticism. Older students are expected to have lower values in these scales.

### 3.5. Beck’s Depression

The percentages of students in each category of BDI are given in Table 9. The majority of the students (79.7%) do not suffer from depression according to BDI. However, 7.7% of the students have borderline clinical depression and 9.4% have moderate depression. Treating the scale as binary scale (depression/no depression) we examine its relations to the students’ characteristics and present the results in Table 10. The logistic regression model is adjusted to age and gender.

**Table 9:** Percentage of students in each category.

Beck Depression Inventory (BDI)	
No depression	509 (79.7%)
Borderline clinical depression	49 (7.7%)
Moderate depression	60 (9.4%)
Severe or extreme depression	21 (3.3%)
Total	639

**Table 10:** Logistic regression coefficients for Beck Depression Inventory (BDI).

Demographic	OR	*p*-Value
**School**		<0.001
Law	1.12	0.792
Health	0.24	0.002
Science	0.26	<0.001
Other	1.01	0.992
**Residence**		<0.001
Students’ residence	0.22	<0.001
Other	0.56	0.081
**Health problem**		
Yes	2.07	0.057
**Traumatic death**		
Yes	2.64	0.002

It turns out that students who are studying at Health and Science schools have lower probability of being diagnosed with depression than students in school of Philosophy. In particular, they have almost 75% lower odds of having depression than students in Philosophy, while all the other variables remain constant.

Depression is also related to the residence. Students who are staying in students’ dormitory or somewhere else have lower probability of having depression than those who are staying with their families, given that they have the same gender, age and all the other variables are the same. Students who have health problems are twice as likely to have depression as the students who do not have health problems. Students who have experienced a traumatic death in their families have higher odds of having depression than those who do not have such experience while the remaining variables are the same.

## 4. Discussion

The aim of this study is to explore the psychometric characteristics of the Greek college students. In an analysis of the results, it turns out that state and trait anxiety scales vary among the school students enrolled, their origin and health status. In addition, students from the school of Health science are expected on average to have lower scores on STAI scales than students from all other departments and students who have health problems are expected to have higher score on STAI scales. Heaman [26], in a study which examined the effects of a 5-week stress management program for 40 junior baccalaureate students, found a significant reduction in state anxiety, while the state anxiety of the control groups remained relatively unchanged. Kawamoto *et al.* [27], in a study aiming to analyze the educational effect and the factors of psychological stress of bedside practice on psychiatric nursing students using the STAI-test, showed that the nursing students frequently complained about anxiety before the bedside practice because they had only studied about the psychoses. Brown *et al.* [28] in a study evaluating the effects of a single session of exercise and quiet rest on blood pressure and state anxiety response of physically challenged college students found that there was a significant decrease in state anxiety.

Exploring the results from the EPQ scale psychoticism is significantly related to the students’ origin, school, family status and semester. Students in the schools of Law, Economics and Political Science and in Science have significantly higher odds than the students in Philosophy and students who are studying for more than four years have higher odds than those who have not completed the 4th year of their studies.

The subscale of neuroticism is significantly related to the students’ school and the variables indicating whether they have their own income and have visited the psychiatric unit in the past. Students who are studying Science have lower probability of scoring high in neuroticism than students in Philosophy. Students who have their own income have higher odds of giving positive answers than students without any income. Besides that, students who have visited the psychiatric unit in the past have 31% higher odds of giving positive answers than the students who have not visited the unit in the past. It turns out that males have significantly lower odds than females (deleted).

The subscale of extraversion is significantly related to the students’ family psychiatric history and whether they are working or not. Students who are working have higher odds of scoring higher in this scale than students who are not working. Finally, older students tend to have lower odds than younger students. Grance *et al*. [29] found that certain personality characteristics such as sensation seeking, impulsivity, aggressiveness and extraversion are associated with alcoholism.

The subscale of lie is significantly related to the students’ residence, their addiction to drugs and the occurrence of a traumatic death in their family. Students who are staying in students’ dormitory have almost 50% higher odds of giving positive answers to the questions related to the lie scale than students who are staying with their family. Students who have other residence (including staying with brother/sisters, flat mates or alone) have 24% higher odds of giving positive answers than the students who are staying with their family. Students who are addicted to drugs have lower odds than students who are not drug addicts.

Students who originally come from Attica have, on average, higher scores in somatization, phobic anxiety and paranoid ideation than the other students. Students from abroad have, on average, higher scores in interpersonal sensitivity and psychoticism than students who hail from other parts of Greece. The mean scores of somatization, interpersonal sensitivity and depression of the students from the school of Philosophy are much higher than the other students. The students from Philosophy, Science and other departments score considerably higher on the scales of obsessive compulsive, phobic anxiety, paranoid ideation and psychoticism than the students in Health school.

Zhang *et al.* [30] in a sample of Chinese college students found that the depression, somatization, obsessive-compulsive and phobic anxiety subscales of SCL-90 affect the university students and focused on the sport competition.

Hu *et al.* [31] investigated using the SCl-90the suicidal ideation among Chinese college students and discovered that one year prior to the investigation, 14.6% of respondents had suicide ideation, 2.5% had made a specific suicide plan, and 1.8% had made a suicide attempt. The main risk factors for suicide ideation were dissatisfaction with the selected major of study, limited social support, recent negative life events and depressive tendency. Jackson *et al.* [32], exploring the associations between psychological symptoms assessed by the Symptom Check List-90 and loneliness in college students, found a significant association between loneliness and interpersonal sensitivity and depression while Keane [33] administered in 42 medical students the Opinion About Mental Illness (OMI) questionnaire and Symptom Checklist (SCL-90-R) and found out that the students’ own psychological distress did not have an effect on attitude change. Luo *et al*. [34], exploring, in a study, the factors affecting 288 college nurse students’ psychological status, and the interactions between mental symptoms and stressful factors, found positive correlations between stressful events and the total score of SCL-90.

The majority of the students (79.7%) do not suffer from depression according to the Beck’s depression inventory scale. However, 7.7% of the students have borderline clinical depression and 9.4% have moderate depression. Students who are staying in students’ dormitory or somewhere else have lower probability of having depression than those who are staying with their families, given that they have the same gender, age and all the other variables are the same. Students who have health problems are twice as likely to have depression as the students who do not have health problems. Students who have experienced a traumatic death in their families have higher odds of having depression than those who do not have such experience while the entire remaining variables are the same. Catanzaro *et al.* [35] in astudy administered the Beck Depression Inventory (BDI) in a sample of 1.177 college students. The results show a wide range of scores suggesting that some of these college students reported high levels of dysphoria (32.7%) scored 10 or greater, a commonly accepted cut-off for mild depression in clinical contexts, with 87 subjects (7.4%) scoring at or above the recommended cut-off for moderate depression. Garsia-Villamisar *et al*. [36] in study examined the relationship between eating disorders, depressive mood by using the Beck Depression Scale and perfectionism in female undergraduate Spanish students. The results demonstrated the importance of socially prescribed perfectionism in mediation of the relationship between depressive mood and symptoms of eating disorders. In another study Sing *et al.* [37], aiming to clarify that insomnia exerts a mediating or moderating effect on the optimism-depression association in 529 Chinese college students who completed the Beck Depression Inventory (BDI), found that insomnia qualifies as a mediator, suggesting considerable variance in depressive symptoms of college students could be due to change in their sleep pattern. Moo-Estrella *et al.* [38] evaluated the prevalence of depressive symptoms, and possible sleep disturbances in 340 college students using the Beck Depression Inventory and Epworth Sleepiness Scale and found diverse sleep alterations in large proportion of the studied subjects, which were more severe in those who showed depressive symptoms. Finally, Tashakkori *et al*. [39], investigating the factor structure of the Beck Depression Inventory in 405 college students, found that the most general factor seemed to be a measure of helplessness and self-devaluation confirming the usefulness of the BDI as a measure of depression. This study aims to examine the psychometric properties of the Greek students in the initial assessment and felt that the approximation of individual elements of psychopathology, like sleep disorders or the use of drugs or alcohol in the college student population, could clarify their psychosocial needs.

## 5. Conclusions

This study aims to examine the psychometric properties of the Greek students in the initial assessment and felt that the approximation of individual elements of psychopathology, like sleep disorders or the use of drugs or alcohol in the college student population, could clarify their psychosocial needs. Anxiety, somatization, personality traits and depression are related with the students’ college life.
